# The Effectiveness of Live and Prerecorded Video Demonstrations in Teaching Restorative Dentistry to Undergraduate Students: Cohort Study

**DOI:** 10.2196/74383

**Published:** 2025-09-25

**Authors:** Rana Alkattan, Lulwah Alreshaid

**Affiliations:** 1 Department of Restorative and Prosthetic Dental Sciences, College of Dentistry, King Saud bin Abdulaziz University for Health Sciences, 7111-Al Rimayah District, Riyadh, 14611, Saudi Arabia, 966 554450818; 2 Ministry of National Guard Health Affairs, King Abdullah International Medical Research Center, Riyadh, Saudi Arabia; 3 Ministry of the National Guard - Health Affairs, King Abdulaziz Medical City, Dental Services, Riyadh, Saudi Arabia

**Keywords:** dental education, prerecorded video, live demonstration, restorative dentistry, gender differences

## Abstract

**Background:**

Mastering complex psychomotor skills is essential in undergraduate dental education; however, traditional live demonstrations (LDs) face limitations such as high instructor-to-student ratios and restricted viewing angles. Prerecorded video demonstrations (VDs) offer scalable, repeatable instructions and the ability to integrate multimedia cues but may lack real-time interaction and immediate feedback. There is limited evidence comparing these teaching modalities, particularly regarding gender differences, in the acquisition of restorative dentistry skills.

**Objective:**

This study aimed to (1) compare first-year dental students’ knowledge acquisition and procedural performance following a LD versus a prerecorded VD of a class II amalgam restoration and (2) evaluate whether gender influences outcomes within each demonstration method.

**Methods:**

A total of 51 students enrolled in an Introduction to Operative Dentistry course (2024‐2025) participated in this cohort study. The students were randomized into 2 groups: LD (26/51, 51%) or VD (25/51, 49%). Both groups received identical lectures and demonstrations of a standardized class II cavity preparation and amalgam restoration. Knowledge was assessed via preprocedural and postprocedural multiple-choice questionnaires, and the procedural performance was graded by 2 blinded raters using a 10-point rubric. Student perceptions were measured with an 8-item Likert survey. Mixed ANOVA and independent and paired 2-tailed *t* tests evaluated between-group and within-group differences, while gender analyses used factorial ANOVA. Interrater reliability (interclass correlation coefficient=0.991) was confirmed.

**Results:**

The baseline knowledge scores did not differ between the 2 groups. After the demonstration, knowledge was significantly higher with LD (mean 71.22, SD 17.3) than VD (mean 58.4, SD 21.7; *P*=.02; Cohen *d*=0.65). The LD method demonstrated significant within-group improvement (*P*<.001; Cohen *d*=0.83). Procedural grading favored LD (mean 8.3, SD 0.9 vs mean 7.9, SD 1.0); however, results were not statistically significant (*P*=.08; Cohen *d*=0.50). No significant differences were found in the student perception survey. Gender analysis revealed that male students in the LD group achieved higher postknowledge scores (mean 74.0, SD 12.3 vs mean 55.0, SD 24.3; *P*=.03), greater score improvements (*P*=.03), and higher grading scores (mean 8.5, SD 0.6 vs mean 7.6, SD 1.3; *P*=.03) compared to those in the VD group. No significant differences were observed among female students.

**Conclusions:**

LDs yielded superior knowledge acquisition and better performance compared to VDs, particularly for male students. VDs remain a viable alternative when supplemented with interactive elements and instructor feedback. Blended teaching models integrating live and video methods may optimize the demonstration experience for the students, thus enhancing the learning outcomes.

## Introduction

### Background

Dental undergraduate education involves learning the science and practice of dentistry, culminating in the mastery of complex procedures that require refined manual skills. This is accomplished through didactic and laboratory-based training, emphasizing the development of intricate psychomotor skills for ultimately performing such procedures in a clinical environment [1]. Dental procedures can be complex and cannot be conveyed using only text and images in presentation formats. Hence, they are often taught by visual observation of the intended procedure in a simulated clinical environment. This typically involves a live, step-by-step demonstration by an instructor to a group of students; thereafter, the students are expected to perform the procedure independently [2].

Traditional live demonstrations (LDs) pose limitations in effectively conveying these skills. LDs offer real-time observation but face challenges such as a high instructor-to-student ratio limiting personalized attention and a limited view of the procedure from a student’s perspective due to positioning within the simulated environment [3]. On the other hand, prerecorded video demonstrations (VDs) can present a potential alternative. Their advantages include scalability [4], wherein a wider student audience can access the content, and enhanced realism [5], as the procedures can be performed inside a mannequin head, replicating the clinical setting. However, the reduced interaction between the student and the instructor and the limited opportunity for student-instructor dialogue and real-time clarification and discussions may make students feel disconnected [6]. Furthermore, VDs are also typically filmed from an angle that may be different than the instructor’s or students’ perspective [7]. Hence, they require a trained instructor skilled in performing procedures while maintaining camera focus and addressing student concerns. Nonetheless, most studies do not describe sufficient information on how a video is presented.

Amid the growth of computer-based e-learning, traditional teaching methods alone may not meet the educational needs of the new generation of dental students [8]. With technological advancement, high-resolution videos showing each step of a procedure from different angles have become possible [9]. The ability to embed texts and images into the recording is also an advantage not offered by LDs [10]. Furthermore, the accessibility of these videos allows students to review specific steps repeatedly at their own pace without the need for instructor dependency. Students may replay any part of the video outside of scheduled classroom hours and have the video stream as they simultaneously perform the procedure, fostering learner autonomy [11]. This method may not only reduce educational costs but also enable teaching anytime and anywhere, shifting the process from teacher-centered to student-centered learning.

While e-learning technologies are increasingly used in undergraduate dental education, limited research exists comparing traditional and modern methods for practical skill development, particularly in restorative dentistry. In dental education, comparing LDs with VDs is important due to ongoing challenges in faculty availability and the need for efficient teaching methods. LDs, while valuable, demand significant faculty time and may vary in delivery between instructors, whereas VDs can help address the faculty shortage by allowing content to be reused across multiple cohorts. Videos also ensure standardization of instruction, providing every student with the same sequence and detail of a procedure. Furthermore, in large classes, videos enhance accessibility and scalability, ensuring all learners have equal opportunities to observe critical steps. By directly comparing both methods, educators can better evaluate learning outcomes, student performances, and cost-effectiveness, guiding decisions on the most effective blend of approaches for dental training. Comparing LDs with VDs among genders is also insightful in evaluating potential differences in learning style preferences and confidence or participation levels. Studies suggest that male and female students may engage differently with instructional formats, with some students benefiting more from interactive, real-time learning, while others prefer the ability to review material independently at their own pace [12]. In addition, LDs can sometimes feel intimidating, limiting participation among students who are less comfortable asking questions in front of peers, whereas VDs provide a private, repeatable way to observe and learn [13]. Examining these differences can help educators design more inclusive teaching strategies that support all students effectively.

### Objectives

This research can potentially address the development of blended learning approaches, combining the advantages of LDs and prerecorded VDs for optimal learning outcomes in dental skills training [14,15]. Therefore, the aim of this study is (1) to compare students’ performance following hands-on LD of a dental proximal restoration to a prerecorded VD of the same procedure and (2) to compare the knowledge and performance of male and female students within both demonstration groups. We hypothesize that (1) the prerecorded VD will be as effective as the LD and (2) there will be no difference in the knowledge and performance of students based on gender.

## Methods

### Overview

A total of 53 first-year dental students, enrolled in the Introduction to Operative Dentistry course (RSTO 311) at the College of Dentistry, King Saud bin Abdulaziz University for Health Sciences, Riyadh, Saudi Arabia, were invited to participate in this cohort study during the academic year 2024-2025. The sample size was based on the cohort size of the course. The collection and analysis of students’ responses and grades were processed anonymously, and student results were treated confidentially. The students were then quasi-randomly divided into 2 groups based on their student serial number by an independent research assistant not involved in the course: those with odd serial numbers were allocated to LD, while those with even serial numbers were allocated to VD. Allocation was concealed from students and instructors until the start of the intervention. No specific measures were taken to balance gender during randomization.

### Ethical Considerations

Institutional review board ethics approval was obtained from King Abdullah International Medical Research Center for this study (IRB/1044/25). The students were asked to participate voluntarily after a signed written consent form was obtained, with the option to withdraw from the study at any time. The collection and analysis of students’ responses were hanlded securely. All responses and grades were processed anonymously and student results were treated with respect and confidentiality.

### Study Design

The study was performed in the context of a preclinical course for restorative dentistry, Introduction to Operative Dentistry. This year-long course is held once a week and serves as students’ first exposure to restorative procedures. It is given in a simulation laboratory where the students are asked to perform different procedures on plastic model teeth (Nissin Dental Products Inc) mounted in the mannequin heads of the simulation units (Planmeca Compact iSim, Planmeca). The students begin the course by learning basic theoretical and practical knowledge on cavity preparations for dental amalgam. Before this study was conducted, all students had previously performed 7 class I cavity preparations and amalgam restorations on upper and lower posterior teeth. This study entailed the performance of their first class II cavity preparation involving the occlusal and mesial surfaces of a mandibular right first molar, proper Tofflemire matrix placement, and amalgam restoration.

Each step of the planned procedure was demonstrated in either of the 2 methods: LD or VD. The dental simulation laboratory comprised 2 rooms, in which the demonstrations were delivered in 2 parallel groups simultaneously, that is, 1 group in each room. The LD included a 30-minute live hands-on demonstration by 1 of the 3 restorative dentists to a group of students, with an instructor-to-student ratio of 1:6, during which both the instructor and the students could comment on the procedure and ask questions. The demonstrations were standardized by having all demonstrators view the prerecorded video given to the VD group, with a checklist outlining the sequence of steps of the intended procedure to ensure that the demonstrators delivered the procedural steps in a uniform and comparable manner. The VD included a prerecorded video of the same procedure performed by a single demonstrator, with a total duration of approximately 30 minutes, shown on individual screens at each student’s station. Both the LD and the VD described identical steps involved in preparing and restoring a class II amalgam.

**Figure 1.**
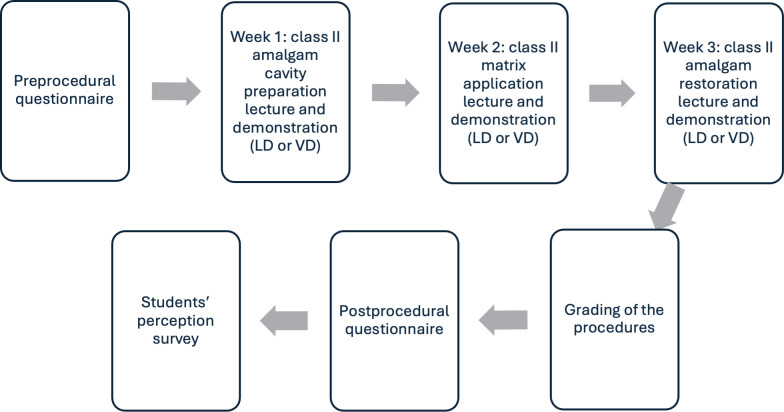
Workflow of the study comparing live demonstration (LD) with video demonstration (VD) in first-year dental students.

The students participated in the study for 3 consecutive weeks, from February 2025 to March 2025, as part of their regular weekly schedule as per their RSTO 311 course curriculum. The course is given once a week, with 3 sessions correspondingly divided as follows: week 1 (class II amalgam cavity preparation lecture and demonstration), week 2 (class II matrix application lecture and demonstration), and week 3 (class II amalgam restoration lecture and demonstration). Each lecture was given by a single faculty member immediately before the assigned corresponding laboratory session. A flowchart of the study design is shown in Figure 1.

The students first received a preprocedural questionnaire to assess their knowledge before the lecture and demonstration, consisting of 10 four-choice questions about different procedural steps (Multimedia Appendix 1).

The students were then randomly divided into 2 parallel groups: LD or VD. Two blinded instructors not involved in the demonstration then graded the procedure for all students from both groups, and the average grade was recorded. A 10-point rubric was used to grade the procedural steps, with a maximum possible score of 1 for each criterion (Multimedia Appendix 2). The students were expected to perform the procedure after applying the rubber dam; however, no grade was given for the rubber dam placement. Next, the students received a postprocedural questionnaire to assess their knowledge after the lecture and demonstration, consisting of the same 10 four-choice questions used in the preprocedural questionnaire (Multimedia Appendix 1).

Feasibility was evaluated based on the ability to deliver the intervention within regular course sessions. Students’ screens in the VD group were tested before the sessions for any technical difficulties. Acceptability was assessed via an 8-item survey, adapted from a study by Alqahtani et al [15], which was used to measure the students’ perception toward the 2 demonstration methods by capturing students’ satisfaction, perceived clarity, and confidence in performing the procedure. The survey was based on a Likert scale with 5 response options (1=strongly disagree, 2=disagree, 3=uncertain, 4=agree, and 5=strongly agree).

### Statistical Analyses

Mixed ANOVA was used to assess students’ knowledge scores from the preprocedural and postprocedural questionnaires (within groups) as well as the difference between groups. Simple main effects with Bonferroni corrections were used to evaluate the differences between groups at each time point as well as changes within each group. Before and after the demonstration, students’ knowledge was scored as follows: correct answers received a 1-point score, while erroneous answers received a 0-point score. The validity of the questionnaire was confirmed using the content validity method, which included the opinions of a 5-member expert panel. For the grades achieved for the procedure, mean differences between the groups were assessed using an independent samples *t* test. Two instructors independently used the 10-point evaluation form to assess students’ performance. Interrater reliability was measured using the interclass correlation coefficient (ICC) with a 2-way random effects model for absolute agreement ICC (2,1 [indicating a 2-way random, single measure]), as described by McGraw and Wong [16]. The ICC value was 0.991 (95% CI 0.984‐0.995), indicating excellent reliability and consistency in ratings, based on the benchmarks proposed by Cicchetti [17], where ICC values >0.75 are considered excellent. For analysis purposes, the average value of the 2 evaluation scores was used. Gender differences were assessed by factorial ANOVA. While gender stratification was not performed, the randomization process and the relatively balanced gender composition support the internal validity of the study. Dependent variables were assessed for normality using histograms and the Shapiro-Wilk test. A significance level of 0.05 was used for inferential analysis, with *P* values <.05 reported as statistically significant. Statistical analysis was conducted using the SPSS (version 29; IBM Corp).

## Results

### Student Enrollment

**Figure 2.**
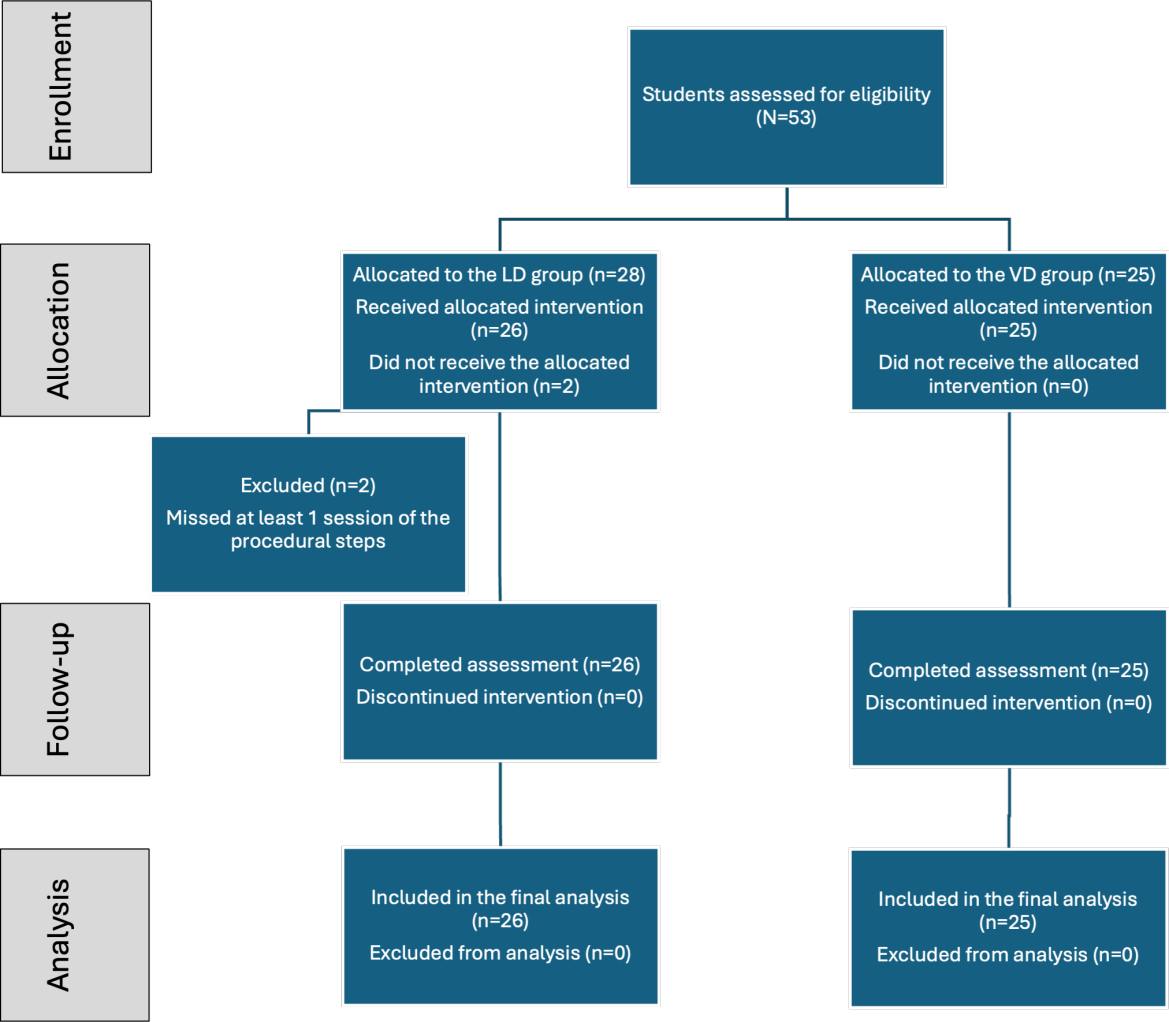
CONSORT (Consolidated Standards of Reporting Trials) flowchart illustrating the flow of students through each stage of the randomized study, including enrollment, allocation, follow-up, and analysis. LD: live demonstration; VD: video demonstration.

A total sample of 51 dental students (male students: 22/51, 43.1%; female students: 29/51, 56.9%) agreed to participate in the study. Two male students from the LD group were excluded because they missed at least 1 session of the procedural steps in which an LD was given following the CONSORT (Consolidated Standards of Reporting Trials) guidelines (Figure 2). The students were allocated into 2 groups: LD (26/51, 51%) or VD (25/51, 49%).

### Predemonstration and Postdemonstration Questionnaires

While this quasi-randomization approach may have allowed for hidden biases related to admission order or other systematic factors, the relatively balanced gender composition and prior procedural experiences helped mitigate potential confounding effects. No significant difference was found between the 2 groups in terms of knowledge scores in the predemonstration questionnaire, suggesting that randomization was successful and the 2 study groups were similar in their preintervention characteristics. However, significantly higher postdemonstration knowledge scores were found with LD compared to VD (*P*=.02). The difference represents a medium-large effect size (Cohen *d*=0.65). Within-group analysis showed significant improvement in knowledge scores with LD (*P*<.001) with a large effect size (Cohen *d*=0.83; Table 1).

**Table 1.** Predemonstration and postdemonstration questionnaire scores and procedural grading scores between live demonstration (LD) and video demonstration (VD) groups.

	LD^a^ (n=26)	VD^b^ (n=25)	Comparison between groups		
			*t* test (*df*)	*P* value	Cohen *d*
Questionnaire scores					
Predemonstration questionnaire, mean (SD)	53.4 (21.9)	52.4 (20.1)	0.18 (49)	.86	0.05
Postdemonstration questionnaire, mean (SD)	71.2 (17.3)	58.4 (21.7)	2.32 (49)	.02	0.65
Grading scores, mean (SD)	8.3 (0.9)	7.9 (1.0)	1.80 (49)	.18	0.50

a Within-group comparison, paired samples *t* test: *F*_1,49_=16.85; *P*<.001; Cohen *d*=0.83.

b Within-group comparison, paired samples *t* test: *F*_1,49_=1.86; *P=*.18; Cohen *d*=0.27.

### Procedural Grading Scores

Procedural grading scores were also found to be higher in the LD group compared to the VD group, but the difference was not statistically significant (*P*=.18) and showed a medium effect size (Cohen *d*=0.50). The results are shown in Table 1.

### Survey Question Scores

Table 2 shows that no significant differences were found between the 2 groups in responses to any of the 8 Likert scale survey questions.

**Table 2.** Survey question scores between the live demonstration (LD) and video demonstration (VD) groups.

Survey questions	LD (n=26), mean (SD)	VD (n=25), mean (SD)	Comparison between groups		
			*t* test (*df*)	*P* value	Cohen *d*
Question 1: “I felt stressed when performing the procedure.”	2.5 (1.3)	2.6 (1.4)	0.16 (49)	.87	0.05
Question 2: “It was easy to perform the procedure.”	3.5 (1.1)	3.5 (1.0)	0.06 (49)	.95	0.02
Question 3: “I was satisfied with my performance in the procedure.”	3.6 (1.1)	3.8 (1.1)	0.87 (49)	.39	0.24
Question 4: “The teaching method was helpful to perform the procedure.”	4.1 (0.9)	3.9 (0.9)	0.59 (49)	.56	0.17
Question 5: “The teaching method was adequate for performing the procedure.”	4.2 (0.8)	3.8 (1.1)	1.41 (49)	.16	0.40
Question 6: “The steps in the teaching method were presented in a clear fashion and easy to understand.”	4.0 (1.0)	4.3 (0.8)	0.93 (49)	.36	0.26
Question 7: “I prefer the teaching method that I have received over the other teaching method.”	3.3 (1.2)	3.8 (1.2)	1.75 (49)	.09	0.49
Question 8: “The teaching method needs further improvement to support my learning.”	3.4 (1.2)	3.4 (1.2)	0.04 (49)	.97	0.01

### Gender-Based Analysis

Table 3 demonstrates that no significant differences were found in the predemonstration knowledge scores between the LD and VD groups between the genders. However, significant differences between the LD and VD groups were found among male students regarding postdemonstration knowledge scores (*P*=.03). The LD group showed higher knowledge scores compared to the VD group (*P*=.03; Cohen *d*=0.93, a large effect size). Similarly, the LD group showed more improvement in knowledge scores from before the demonstration to after the demonstration compared to the VD group (*P*=.03; Cohen *d*=0.94, a large effect size). Male students also showed significantly higher grading scores with LD training compared to VD (*P*=.03; Cohen *d*=0.84, a large effect size). However, gender-based analyses were exploratory due to limited sample sizes; interaction terms were included to test for moderation effects, though power to detect small effects was constrained.

**Table 3.** Postdemonstration knowledge scores, improvement in knowledge scores, and procedural grading scores between live demonstration (LD) and video demonstration (VD) groups between genders.

	LD	VD	Comparison between groups		
			*F* test (*df*)	*P* value	Cohen *d*
Predemonstration knowledge scores^a^					
Female, mean (SD)	55.0 (23.1)	51.5 (22.7)	0.19 (1, 47)	.67	0.15
Male, mean (SD)	51.0 (20.8)	53.3 (17.8)	0.07 (1, 47)	.80	0.12
Postdemonstration knowledge scores^b^					
Female, mean (SD)	69.4 (19.1)	61.5 (19.5)	1.13 (1, 47)	.29	0.41
Male, mean (SD)	74.0 (12.3)	55.0 (24.3)	5.03 (1, 47)	.03	0.93
Knowledge scores improvement (change from predemonstration scores to postdemonstration scores)^c^					
Female, mean (SD)	14.4 (22.5)	10.0 (20.0)	0.28 (1, 47)	.60	0.20
Male, mean (SD)	23.0 (19.2)	1.7 (25.2)	5.12 (1, 47)	.03	0.94
Grading scores^d^					
Female, mean (SD)	8.2 (1.1)	8.1 (0.6)	0.20 (1, 47)	.66	0.18
Male, mean (SD)	8.5 (0.6)	7.6 (1.3)	4.76 (1, 47)	.03	0.84

a Comparison between genders for LD: *F*_1,47_=0.22; *P*=.65; Cohen *d*=0.18. Comparison between genders for VD: *F*_1,47_=0.04; *P*=.84; Cohen *d*=0.09. Gender×group interaction effect: *F*_1,47_=0.23; *P*=.64.

b Comparison between genders for LD: *F*_1,47_=0.34; *P*=.57; Cohen *d*=0.27. Comparison between genders for VD: *F*_1,47_=0.68; *P*=.41; Cohen *d*=0.30. Gender×group interaction effect: *F*_1,47_=0.99; *P*=.33.

c Comparison between genders for LD: *F*_1,47_=0.95; *P*=.34; Cohen *d*=0.40. Comparison between genders for VD: *F*_1,47_=0.89; *P*=.35; Cohen *d*=0.37. Gender×group interaction effect: *F*_1,47_=1.84; *P*=.18.

d Comparison between genders for LD: *F*_1,47_=0.67; *P*=.42; Cohen *d*=0.35. Comparison between genders for VD: *F*_1,47_=1.19; *P*=.28; Cohen *d*=0.42. Gender×group interaction effect: *F*_1,47_=1.82; *P*=.18.

## Discussion

### Principal Findings

The main objective of this study was to evaluate the effectiveness of prerecorded VD compared to LD in teaching class II amalgam to first-year dental students. The findings of this study revealed that while both methods improved student knowledge and performance, LD was associated with significantly higher postdemonstration knowledge scores and higher procedural grading scores (Table 1). Therefore, the first null hypothesis was rejected. The second objective of this study was to evaluate differences in the knowledge and performance of students based on gender. The results showed that although there were no statistically significant differences between genders, male students achieved significantly higher postdemonstration knowledge scores with LD (vs VD), as well as higher procedural grading scores (Table 3), rejecting the second null hypothesis.

The significantly higher postdemonstration knowledge scores in the LD group (Table 1) suggest that the interactive nature of LDs may enhance the understanding and retention of procedural steps. The large effect size (Cohen *d*=0.65) observed further highlights the impact of direct instructor-student engagement on knowledge acquisition. The interactive environment provided by LD allows students to ask questions, clarify doubts, and receive immediate corrections, reinforcing learning and reducing misconceptions. Additionally, observing the instructor’s technique enhances visual learning and provides a deeper understanding of the procedural steps. This aligns with cognitive learning theories emphasizing active participation and feedback as critical components for knowledge retention [18]. Furthermore, LD fosters a collaborative learning atmosphere, where peer discussions and shared experiences can further solidify understanding.

In contrast, the VD group demonstrated less improvement in knowledge scores (Table 1). Although the prerecorded video allowed students to review the material at their own pace, the lack of in-person clarification and the potential for disengagement may have limited their learning outcomes. Moreover, VD students may struggle with passive learning, where attention can fade over time without active engagement. To tackle these challenges, incorporating interactive elements such as quizzes, guided reflections, and discussions could enhance the effectiveness of the VD method. In addition, providing access to instructors for follow-up questions or clarification can help bridge the gap between the benefits of live interaction and the flexibility of video-based learning.

The procedural grading scores showed higher performance in the LD group (Table 1), although the difference was not statistically significant. This finding may be attributed to the hands-on guidance during LDs, enabling students to receive immediate corrections and refine their techniques. Nonetheless, the medium effect size (Cohen *d*=0.50) observed indicates that VD may still be a viable alternative for developing the needed skills, especially when combined with supplementary support, such as follow-up instructor feedback sessions. For instance, integrating video-based learning with practice sessions and virtual simulations can help reinforce muscle memory and improve technique [19]. Additionally, structured peer feedback and self-assessment tools can enhance learning outcomes and provide opportunities for skill refinement [20].

It is also worth noting that VD allows students to pause, rewind, and rewatch the demonstration, enabling repetitive practice and gradual skill acquisition. This flexibility is particularly beneficial for students who need extra time to master specific techniques, prefer self-paced learning, or were unable to attend the class.

The survey results indicated no significant differences between the groups in perceived ease of performing the procedure, performance satisfaction, and clarity of teaching methods. However, the VD group tended to prefer their assigned method over LD (Table 2). Individual learning styles and personal comfort with technology may influence the preference for the VD method. For instance, visual and auditory learners may find video-based content more appealing, and students who experience anxiety in live settings may feel more at ease [21].

While male students in the LD group showed higher postdemonstration scores and procedural grades compared to VD, the nonsignificant group×gender interactions indicated that these findings should be interpreted cautiously. Although the small subgroup sizes limit the generalizability of gender-specific conclusions, significant differences between the LD and VD groups were evident among male students, with the LD method leading to higher postdemonstration knowledge scores and more significant improvements from pre to postdemonstration scores. Male students also performed significantly better in the LD group, as demonstrated by their grading scores (Table 3). However, no significant differences were found among female students, indicating that both demonstration methods were equally effective for this group.

### Comparison With Previous Work

The significantly higher postdemonstration knowledge scores in the LD group compared to the VD group suggest that LD allows the instructor to engage with the students through questions, answers, and discussions, ensuring they understood each step in the presented treatment. This finding is consistent with previous studies emphasizing the benefits of real-time interaction and immediate instructor feedback to aid in concept visualization and maintain student concentration [2,3,22]. Schlafer et al [3] compared hands-on LDs with video-supported demonstrations for teaching aesthetic composite restorations. Their method involved guiding one group of students through a live, hands-on demonstration, while the other group watched a prerecorded video of the same procedure, with the instructor pausing to ask and answer questions and initiate discussions in both groups. Although they did not assess the students’ theoretical knowledge using any test or grade the restorations performed by the students, they found that the video group showed more engagement during in-class discussions and also discussed patient-related aspects more than the hands-on group. Another study by Ramlogan et al [14] similarly compared LD and VD for teaching clinical periodontology to undergraduate dental students. Their results showed that students achieved similar pretest knowledge scores in both groups; however, the mean posttest scores were significantly greater for the live lecture group. Surprisingly, more students favored VDs over live lectures, suggesting the potential of videos as an educational technology to complement live teaching and serve as a means for reaching teaching and learning goals.

In contrast, the VD group in this study demonstrated less improvement in knowledge scores. While VDs offer benefits like enhanced visualization and flexibility, they can also create a detachment from the clinical setting and reduce direct interaction between students and instructors, potentially hindering immediate feedback and personalized guidance. This finding aligns with published literature indicating that video-based learning can be effective but requires additional support mechanisms to maximize student engagement and comprehension [9,23,24]. Rystedt et al [9] highlighted the significance of the reciprocal relationship between visualization, instruction, and interaction in video-based demonstrations, which ultimately contributes to an effective learning experience for students. First, they explained that visualization refers to allowing students to witness treatments from the dentist’s perspective, offering a detailed and magnified view of intricate procedures. Second, they emphasize that the teacher’s instruction is key to guide students’ attention to critical aspects of the treatment, explain the reasoning behind decisions, and connect the demonstration to theoretical concepts. Lastly, they emphasize the need to incorporate an interactive setting with video-based demonstrations, including discussions between teachers and students, fostering student engagement and facilitating a deeper understanding of the material. The shared visual field created by the video can allow for focused discussions and timely clarifications, which enhances the learning process.

Students’ prior experience with multimedia learning, their familiarity with technology, and their baseline motivation can all influence how well they learn from VDs compared to LDs. These factors can affect their engagement; understanding; and, ultimately, their learning outcomes. Students who are already comfortable with learning through videos and other multimedia formats may adapt more quickly to VDs in dental education [25]. Furthermore, students who are already highly motivated to learn may be more likely to engage with both LD and VD. However, students who are less motivated might benefit more from the interactive aspects of LDs, which can provide a more engaging and stimulating learning experience [26]. These factors can influence a student’s ability to understand and retain information as well as develop practical skills. For example, a student who is comfortable with multimedia learning might grasp complex dental procedures more quickly from a VD, while another student might need the hands-on experience of an LD to fully understand the nuances of the technique [27].

While there were no significant differences observed between the groups in how easy they found the procedure, how satisfied they were with their performance, or how clear the teaching methods were, survey results demonstrated that the VD group expressed a tendency to prefer their learning method over the LD group. This finding suggests that some students may value the flexibility and accessibility of video-based learning, even if it does not lead to superior performance outcomes. This is supported by the self-determination theory, which highlights the importance of autonomy in enhancing motivation and learning outcomes [28]. Furthermore, a previous meta-analysis similarly indicated that students with control over their learning pace and environment report higher satisfaction and engagement [29]. A similar study by Alqahtani et al [15] found that while a procedural video for fabricating an orthodontic Adams clasp was as effective as an LD in terms of mean scores in a laboratory exercise, students rated the video higher in perceived clarity and ease of understanding. This may be because the procedural video offered better visualization of the laboratory steps and allowed students to review the procedure independently before, during, or after the laboratory sessions, free from crowding.

The study’s findings also suggest that LDs’ interactive and dynamic style may be particularly beneficial for male students. Published literature indicates that male students tend to prefer hands-on, experiential learning environments that provide immediate feedback and active engagement [30]. The opportunity to ask questions and receive in-person guidance during LD may cater to these preferences, enhancing their learning outcomes [31]. On the other hand, no significant differences were found among female students, indicating that they performed equally in both LD and VD groups. Previous studies demonstrate that female learners may excel in self-paced, independent learning environments, which could explain their equal performance in both groups when compared to male learners [32]. Moreover, female students may demonstrate higher levels of self-regulation and metacognitive strategies, allowing them to overcome the limitations of the VD method [33].

Al-Sbei et al [34] evaluated the impact of integrating a web-based course on the restorative dentistry knowledge of graduate students in Syria, which included theoretical and educational videos. The results showed a significant increase in the participants’ levels of restorative dentistry knowledge after completing the web-based course. This demonstrates that video-based demonstrations may have implications beyond traditional classroom teaching, particularly in cases with difficulty in transportation, lack of qualified places to hold dental courses, and staff shortages. Another study by Kanzow et al [35] evaluated the restorative dentistry knowledge of undergraduate students using screen-captured presentations with narrated audio uploaded onto an online platform during the COVID-19 pandemic. Students viewed the presentations more frequently than they could have attended a conventional lecture and subsequently demonstrated better performance in theoretical knowledge tests in restorative dentistry–related topics compared to other dental specialties. Future research should explore the integration of blended learning approaches that combine the advantages of both LD and VD, such as incorporating interactive video modules and virtual simulations. The opportunity to engage with students under the supervision of an instructor also allows for in-person feedback and error correction, which are crucial for developing psychomotor skills. This aligns with previous findings that emphasize the importance of experiential learning in technical education [36,37].

In this study, gender-based differences warrant further investigation into the underlying factors influencing learning outcomes. Future studies could explore the role of learning preferences, cognitive strategies, and prior experiences, which may influence the effectiveness of different instructional methods and help explain potential gender-related differences. Lastly, it is worth exploring personalized learning styles tailored to individual preferences and learning needs.

### Limitations

The study has several limitations. First, the relatively small sample size may have limited the generalizability of the findings, specifically related to gender analysis, and further research with larger samples is needed to explore potential moderating effects of gender. Second, the study focused on a single dental procedure, and the results may not apply to other aspects of dental education. Third, the study relied on self-reported surveys for student perceptions, which may introduce response bias. Fourth, factors such as prior experience with video-based learning were not controlled, which could influence the study’s outcomes. Lastly, blinding instructors and students was not feasible due to the nature of LD versus VD; however, the raters assessing student performance were blinded to group allocation. This could introduce performance or expectation bias, as students’ motivation or engagement may have been influenced by group assignment. However, to minimize potential bias, all demonstrations were standardized using the same procedural steps, and grading was performed by 2 independent, blinded instructors using a detailed rubric.

### Conclusions

Within the limitations of this study, it can be concluded from the findings that the LD method was more effective in enhancing knowledge acquisition compared to the prerecorded VD method. However, the VD method remains a second-best alternative when needed, particularly when supplemented with interactive elements and instructor feedback. It was also evident that male students benefited more from the LD method than female students, an interesting finding that needs further exploration. Based on the results of this study, students’ demonstration experience can be enhanced by incorporating hybrid learning models. Combining LD with interactive VD modules can improve knowledge acquisition and procedural performance. Furthermore, interactive elements may be added to make the VD method as effective as the LD, such as quizzes, guided reflections or discussions, and instructor feedback to improve engagement and comprehension.

## Supplementary material

10.2196/74383 Multimedia Appendix 1 Preprocedural and postprocedural knowledge questionnaires.

10.2196/74383 Multimedia Appendix 2 Criteria for grading procedural steps.

## Abbreviations

CONSORT: Consolidated Standards of Reporting Trials
ICC: intraclass correlation coefficient
LD: live demonstration
VD: video demonstration
