# Supplementary material for: The Effectiveness of Live and Prerecorded Video Demonstrations in Teaching Restorative Dentistry to Undergraduate Students: Cohort Study
Source: JMIR Form Res. 2025 Sep 25;9:e74383. doi: 10.2196/74383 (PMC12463341; doi:10.2196/74383)
Supplement: Multimedia Appendix 1 [file formative-v9-e74383-s001.docx]

**Appendix 1. Pre and Postprocedural Knowledge Questionnaire**

Q1- In addition to the extent of caries, which of the following determines the correct divergence of the buccal and lingual walls of a class II box?

1. Divergence angle of 45 degrees
2. Until there is enough depth in the gingival seat
3. Enough clearance to allow the passing of the tip of the explorer
4. Half of the bucco-lingual width of the tooth

Q2- In class II preparation, what is the purpose of placing grooves in the buccal and lingual walls of the box at the axiobuccal and axiolingual line angles?

1. To retain the restoration in place
2. To prevent the placed restoration from fracture
3. To make sure all caries and undermined enamel are removed
4. To create enough space for instrumentation

Q3- What is the bur used to create the grooves in axiobuccal and axiolingual line angles in class II preparation?

1. 330 pear-shaped bur
2. 245 pear-shaped bur
3. ½ round-shaped bur
4. ¼ round-shaped bur

Q4- Which of the following hand instruments is only used in Class II cavity preparation?

1. Enamel hatchet
2. Chisel
3. Angel former
4. Gingival marginal trimmer

Q5- Which of the following matrix systems is the ideal choice to restore an MOD Class II cavity for molars?

1. Sectional matrix (pre-contoured band)
2. Retainer-less circumferential matrix
3. Tofflemire matrix
4. Siqveland matrix

Q6- Why placing a wedge with the matrix band is essential during class II amalgam restoration?

1. To protect the adjacent tooth
2. To prevent gingival bleeding
3. To compensate for the band thickness
4. To confine amalgam in place

Q7- To restore a class II cavity preparation. What is the proper sequence of amalgam placement steps?

1. Condensation, carving, burnishing
2. Condensation, pre-carve burnishing, carving, post-carve burnishing
3. Condensation, burnishing, carving
4. Condensation, post-carve burnishing, carving, pre-carve burnishing

Q8- During the amalgam condensation step in class II restoration, which area should you start placing amalgam and then condensing it?

1. Occlusal area
2. Box area
3. Axial grooves
4. Occlusal and box area simultaneously

Q9- What is the key anatomical landmark you should properly restore during class II amalgam restoration?

1. Buccal and lingual grooves
2. Triangular ridges
3. Cusp tips
4. Marginal ridges

Q10- Which of the following hand instruments is helpful in the interproximal carving step of class II amalgam restoration?

1. Hollenback
2. Cleoid discoid
3. IPC
4. PKT no.3
