# Supplementary material for: The Effectiveness of Live and Prerecorded Video Demonstrations in Teaching Restorative Dentistry to Undergraduate Students: Cohort Study
Source: JMIR Form Res. 2025 Sep 25;9:e74383. doi: 10.2196/74383 (PMC12463341; doi:10.2196/74383)
Supplement: Multimedia Appendix 2 [file formative-v9-e74383-s002.docx]

**Appendix 2. Criteria for grading of the procedural steps**

| **Assessment Items** | **Criteria for assessment** | **Grade** |
| --- | --- | --- |
| Cavity Preparation | Adequate cavity depth (pulpal floor) and width (axial wall) | 1 |
|  | Proper outline form with cusp integrity preserved | 1 |
|  | Flat floors and retention form (convergent buccal and lingual walls) achieved | 1 |
|  | Proper proximal Clearance (buccal, lingual and gingival) with no injury to the adjacent tooth | 1 |
| Tofflemire Matrix application | Correct and tight matrix band sealing the gingival floor | 1 |
|  | Proper wedge placement | 1 |
| Amalgam | Sealed margins of the restoration | 1 |
|  | Proper occlusal/embrasure anatomy | 1 |
|  | Adequate proximal contact | 1 |
|  | Smooth surface finish | 1 |
